# Editorial: Why People Gossip and What It Brings About: Motives for, and Consequences of, Informal Evaluative Information Exchange

**DOI:** 10.3389/fpsyg.2020.00024

**Published:** 2020-01-28

**Authors:** Myriam N. Bechtoldt, Bianca Beersma, Maria T. M. Dijkstra

**Affiliations:** ^1^Department of Management and Economics, EBS University of Business and Law, Oestrich-Winkel, Germany; ^2^Department of Organization Sciences, Vrije Universiteit Amsterdam, Amsterdam, Netherlands

**Keywords:** gossip, motives, groups, cooperation, prosocial behavior, antisocial behavior

You do it, we do it, everyone does it: talk about others in their absence. Estimates suggest that in two thirds of their conversations people are talking about others who are not present (e.g., Levin and Arluke, 1985; Dunbar et al., 1997). But people do not talk openly about the fact that they like to gossip. Rather, they claim that they do it less frequently than others (Hartung and Renner, 2013), and they do not like those who are known for it (Farley, 2011; Ellwardt et al., 2012). The negative view of gossip among laypeople contrasts with scientific insights suggesting that gossipers' motivation is fueled more strongly by epistemic motives (i.e., a desire to understand the social environment), or pro-social motives (i.e., a desire to help others), than malicious motives (e.g., Beersma and Van Kleef, 2012). So gossip is a phenomenon similar to the elephant in the room: Everyone knows it is there, but no one talks about it.

This special issue does talk about gossiping: Six papers address various facets of this socially disapproved yet ubiquitous phenomenon. They present a nuanced view of gossip by relativizing both the globally negative view of it among laypersons and more positive scientific perspectives on it. Three papers focus on gossip senders' motivation to engage in, or refrain from, gossiping; three papers focus on the reactions of both gossip recipients and gossip targets, and they also address the longer-term consequences of gossip that had been neglected in previous research. The papers employ different methods: some are based on experimental designs, others used a survey design, and one paper examined the factor structure of a measurement instrument. Finally, one paper is a theoretical paper.

Regarding gossip senders' motives, first, Giardini and Wittek argue that understanding the reasons why people *do not* gossip may provide useful insights. They critically review the gossip literature in order to highlight the conditions under which people might refrain from sharing third-party information. Subsequently, they apply Goal Framing theory to gossip, arguing that most gossip studies illustrate the mechanisms in which the hedonic gratification derived from gossiping is reinforced by gain-related or normative goals. However, these frames can also prevent gossip. Therefore, the authors argue that depending on different configurations of frames and relations between actors, the perceived costs of sending gossip may be far higher than the previous literature suggests.

Second, Hartung et al. confirm that typically people do not gossip to harm others–not even individuals with “dark” personalities. The authors also present a validated German version of the Motives to Gossip questionnaire by (Beersma and Van Kleef, 2012).

A third paper that focuses on gossip senders' motives is the paper by Dores Cruz, Beersma et al.. They report that situational variables can trigger different motives to gossip; having the opportunity to gossip to a potential victim of a norm violator increased the motivation to use gossip to protect others compared to a non-victim. This paper also addresses targets' responses to gossip; findings show that whereas negative gossip about targets' work performance increased their immediate efforts, it lowered their motivation for long-term cooperation with the gossipers. These results nuance earlier positive views on gossiping indicating that gossip educates people to conform to social norms (e.g., Dunbar, 2004; Feinberg et al., 2012).

The paper by Wu et al. also points toward possible negative long-term consequences. They show that gossiping increases individuals' motivation to cooperate both in the dictator game and in the ultimatum game. Although these results confirm the utility of gossip in promoting cooperation, there seems to be a rebound effect, as people who played the ultimatum game and knew that their reputation was communicated to a third person through gossip returned less money to that third person in a subsequent trust game.

Martinescu et al. address the emotional consequences of gossip for its targets. Whereas, targets of positive gossip experienced positive self-conscious emotions, targets of negative gossip experienced negative self-conscious emotions, especially when they had low core self-evaluations. In turn, these negative self-conscious emotions predicted repair intentions. Positive gossip also led to positive other-directed emotions, which predicted intentions to affiliate with the gossiper. Negative gossip, however, generated other-directed negative emotions, especially for targets with high reputational concerns. These negative emotions predicted retaliation intentions against the gossiper. Gossip apparently has self-evaluative and other-directed emotional consequences, which predict how people intend to react after hearing gossip about themselves.

Finally, the paper by Dores Cruz, Balliet et al. is more methodologically focused. This paper aims to “get a grip at the grapevine,” as the title states, by extending the existing Motives to Gossip Questionnaire (Beersma and Van Kleef, 2012) through adding a subscale for emotion venting and by examining whether the underlying factor structure of the scale is robust across different definitions of gossip. Confirmatory factor analysis confirmed the five-factor structure and supported full invariance across three different definitions of gossip.

Together, the papers in this special issue contribute to our knowledge about gossip in several ways. First, they offer new insights into the motives that drive gossip senders' behavior. Whereas, previous studies have often implicitly taken the perspective that gossip is driven by one motive (e.g., group protection or strategic self-interest, see Beersma et al., 2019), this set of studies shows that different motives can drive gossip behavior. Gossip is, thus, neither exclusively motivated by noble motives nor by malicious ones, as previous studies have suggested. Specifically, Dores Cruz, Beersma et al. show that different situations can activate different motives to gossip; Hartung et al. show that not even those with “dark” personalities gossip to harm others, and Dores Cruz, Balliet et al. show that different motives to engage in gossip can be reliably distinguished from one another across different conceptualizations of gossip behavior. Finally, by showing why people would refrain from gossiping, Giardini and Wittek add to this broader perspective on gossip motives.

Second, the studies presented here offer new insights into the consequences of gossip by being among the first that examine outcomes beyond short-term cooperation. Whereas, previous studies have shown that gossip can increase the adherence to cooperative norms in groups (Beersma and Van Kleef, 2011; Feinberg et al., 2012), the papers in this special issue show that gossip can reduce trust in long-term interactions (Wu et al.), have important emotional repercussions for targets (Martinescu et al.) and reduce intentions to cooperate in the long run (Dores Cruz, Beersma et al.).

In conclusion, the studies reported here move away from the existing perspective on gossip as a one-sided, purely negative or purely positive, phenomenon. We hope that the current set of papers forms an inspiration for further studies that will continue to explore gossip as a phenomenon that is driven by different motives and that has both beneficial and detrimental effects for senders, recipients, and targets.

## Author Contributions

MB, BB, and MD contributed to writing this editorial.

### Conflict of Interest

The authors declare that the research was conducted in the absence of any commercial or financial relationships that could be construed as a potential conflict of interest.
